# Aβ42 oligomers modulate β-secretase through an XBP-1s-dependent pathway involving HRD1

**DOI:** 10.1038/srep37436

**Published:** 2016-11-17

**Authors:** Yannis Gerakis, Julie Dunys, Charlotte Bauer, Fréderic Checler

**Affiliations:** 1 Université Côte d’Azur, INSERM, CNRS, IPMC, France, Laboratory of excellence DistALZ, 660 route des Lucioles, 06560, Sophia-Antipolis, Valbonne, France

## Abstract

The aspartyl protease β-site APP cleaving enzyme, BACE1, is the rate-limiting enzyme involved in the production of amyloid-β peptide, which accumulates in both sporadic and familial cases of Alzheimer’s disease and is at the center of gravity of the amyloid cascade hypothesis. In this context, unravelling the molecular mechanisms controlling BACE1 expression and activity in both physiological and pathological conditions remains of major importance. We previously demonstrated that Aβ controlled BACE1 transcription in an NFκB-dependent manner. Here, we delineate an additional cellular pathway by which natural and synthetic Aβ42 oligomers enhance active X-box binding protein XBP-1s. XBP-1s lowers BACE1 expression and activity indirectly, via the up-regulation of the ubiquitin-ligase HRD1 that acts as an endogenous down-regulator of BACE1. Thus, we delineate a novel pathway by which cells could compensate for Aβ42 oligomers production and thus, associated toxicity, by triggering a compensatory mechanism aimed at lowering BACE-1-mediated Aβ production by a molecular cascade involving XBP-1s and HRD1. It thus identifies HRD1 as a potential target for a novel Aβ-centered therapeutic strategy.

Alzheimer’s disease (AD) is a neurological disorder, which is one of the most common dementia among elderly people. One of the main hypotheses regarding AD etiology, called the amyloid cascade hypothesis, considers the Aβ peptide at a central position of a sequence of cellular events leading to clinical picture, dementia and ultimately, death.

The amyloidogenic pathway yielding Aβ^1^,^2^,^3^ involves a rate-limiting cleavage of the β-amyloid precursor protein (βAPP) by the β-site amyloid precursor protein cleaving enzyme 1 (BACE1)^4^, thereby producing a secreted fragment (sAPPβ) and an intramembranous C-terminal fragment (C99), which is then processed by γ-secretase to release Aβ peptide and an intracellular domain, AICD^5^. The definitive nature of the Aβ species (intracellular, truncated, aggregated) that genuinely triggers aversive effects such as oxidative stress^6^, elevated calcium toxicity^7^, mitochondria and cells energy production defects^8^, excitotoxicity of neuronal axons^9^, all of these leading to cell death and apoptosis^10^, remains a matter of discussion.

BACE1 is considered as a key therapeutic target not only because its inhibition precludes Aβ production but also because BACE-1-mediated βAPP cleavage only, generates C99 that had been shown to trigger cellular perturbations and toxicity even in absence of Aβ in mice models of AD^11^,^12^,^13^. BACE1 is highly expressed in neurons and, unlike is the case for γ-secretase, its expression increases during ageing as well as in the brain of AD patients^14^,^15^. This aroused interest for delineating the mechanistic regulation of this enzyme and more particularly, for transcription factors regulating BACE1, some of which also increased with age. These transcription factors, induced by stress and environmental conditions, such as c-Jun^16^, nuclear factor-kappa B (NF-κB)^17^, nuclear factor of activated T-cells 1 (Nfat-1)^18^, specificity protein 1 (Sp1)^19^, Yin Yang 1 (YY1)^20^, signal transducer activator of transcription 3 (STAT3)^21^ and p25/cdk5^22^ have been shown to directly bind BACE1 promoter, to up-regulate BACE1 gene transactivation and, thereby, increase its expression and catalytic activity.

Protein misfolding, accumulation in the endoplasmic reticulum (ER), widely known as ER stress and abnormal protein aggregation have been well documented in AD and are intimately linked to BACE1^23^. ER stress activates the unfolded protein response (UPR), an adaptative sensor-regulator network aimed at restoring the protein folding homeostasis or, in case of irreversible stress damage, responsible for apoptosis activation^24^,^25^. Three main sensors control UPR signaling: Activating Transcription Factor 6 (ATF6), Protein kinase RNA-like Endoplasmic Reticulum Kinase 1 (PERK1) and the endoribonuclease Inositol Requiring Enzyme 1 (IRE1). Upon ER stress signal, IRE1 splices the mRNA of x-box binding protein-1 (XBP-1), thereby yielding a more stable form that is then translated into an active transcription factor (XBP-1s)^26^. Besides its well established function in UPR signaling, XBP-1s transcription factor has been implicated in additional physiological functions including glucose and lipid metabolism control but could also be modulated in neurodegenerative diseases including AD^27^,^28^. Interestingly, XBP-1s was recently shown to regulate memory formation^29^.

Although UPR and XBP-1s activation are generally considered as early neuroprotective responses aimed at limiting Aβ-related neurodegeneration^30^,^31^, the mechanisms by which XBP-1s triggers cellular protective phenotypes have not been yet fully elucidated.

Here, we describe a novel cellular cascade by which synthetic and natural Aβ42 oligomers modulate BACE1 expression and activity through a pathway involving new players, HRD1 ubiquitin ligase and XBP-1. Thus, our study unravels a potential compensatory mechanism by which cells could tune down Aβ-oligomers-associated toxicity.

## Results

### Synthetic and natural Aβ42 oligomers increase BACE1 mRNA expression

We previously established that exogenous application of Aβ42 monomers or transient transfection of cDNA encoding Aβ42 enhanced BACE1 mRNA levels via NFκB^32^. This data was conforted by our demonstration that cells overexpressing Swedish-mutated βAPP and producing supraphysiological levels of Aβ displayed enhanced NFκB-dependent BACE1 transcription and activity^17^. Although these studies consistently indicate an indirect control of BACE1 by its own APP-derived product, it did not definitely demonstrate the capability of Aβ42 oligomers (Aβo), currently recognized as the most toxic Aβ species^33^,^34^ to mimic this effect. Furthermore, it did not examine whether alternative NFκB-independent pathways could govern Aβ-linked control of BACE1.

**Figure 1:**
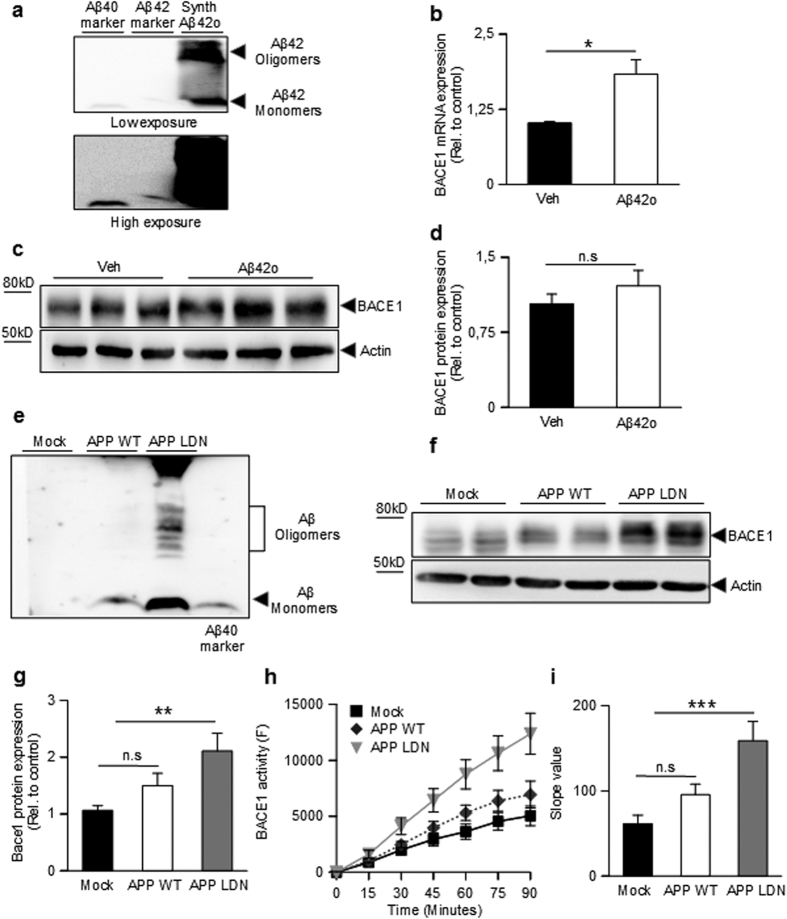
Influence of synthetic and natural Aβ42 oligomers on BACE1. (**a**) Western blot analysis of synthetic Aβ42 oligomers (Aβo) obtained as described in the Methods. (**b**) Quantitative PCR analysis of BACE1 mRNA levels in SH-SY5Y neuroblastoma cells treated overnight with synthetic Aβo. BACE1 mRNAs are normalized using the expression of human Rpl69 housekeeping gene. (**c**,**d**) Western blot analysis (**c**) and densitometric quantification (**d**) of BACE1 expression in Aβo-treated SH-SY5Y cells. Graphs show the mean of three independent experiments (6 and 8 biological replicates per group in (**b** and **d**), respectively). A Student one-tailed T-test is applied for statistics (*p-value = 0,02 and n.s = non significant, in (**b** and **d)**, respectively). (**e**) Western blot analysis of media recovered from CHO cells stably overexpressing either wild type βAPP (APP_WT_) or βAPP bearing the London mutation (APP_LDN_) compared to mock-transfected CHO cells. (**f**,**g**) Western blot analysis (**f**) and densitometric analysis of BACE1 (**g**) protein expression in the indicated cell lines. The graph shows the mean of 5 independent experiments (10 biological replicates per group). For statistics, a one-way ANOVA followed by a post hoc Bonferroni-Holm test is applied (**p-value = 0,0053 alpha 0,01; n.s non significant). (**h**) Specific BACE1 activity measured in the indicated cell line. BACE1 activity corresponds to the inhibitor-sensitive fluorimetry and is expressed relatively to control and normalized by protein quantification using Bradford method. The graph represents the mean BACE1 activity of 4 independent experiments, with 8 biological replicates plotted per group. (**i**) Represents the slope value of the curves in (**h**). Statistic analysis is as in B (***p-value = 0,001 alpha 0,01; n.s non significant).

We examined first the influence of synthetic oligomeric Aβo (Fig. 1a) and we show that they increase BACE1 mRNA levels in SH-SY5Y cells (Fig. 1b) while a trend of enhanced BACE1 protein expression was observed that did not reach statistical significance (Fig. 1c,d). It was unclear whether this apparent dichotomy between Aβo-associated influence on BACE1 mRNA and protein expressions could be accounted for by the experimental procedure where acute exposure to oligomers or by kinetic delay between transcription process and traduction. Thus, we envisioned determining whether naturally occurring Aβo chronically produced by chinese ovary hamster cells (CHO) stably engineered to express either wild type βAPP or βAPP harboring the London mutation (V717I; APP_LDN_)^35^ could modulate BACE1 expression and activity. Figure 1 shows that CHO APP_LDN_ cells indeed yield high levels of Aβo (Fig. 1e) concomitant to increased BACE1 expression (Fig. 1f,g) and enzymatic activity (Fig. 1h,i). Overall, this set of data indicates that both synthetic and naturally occurring Aβo modulate BACE1 mRNA and protein expression and activity although we cannot totally preclude the possibility of a marginal contribution of Aβ monomers.

### Synthetic and naturally occurring Aβ oligomers up-regulate mRNA levels of spliced X-Box binding protein 1

**Figure 2:**
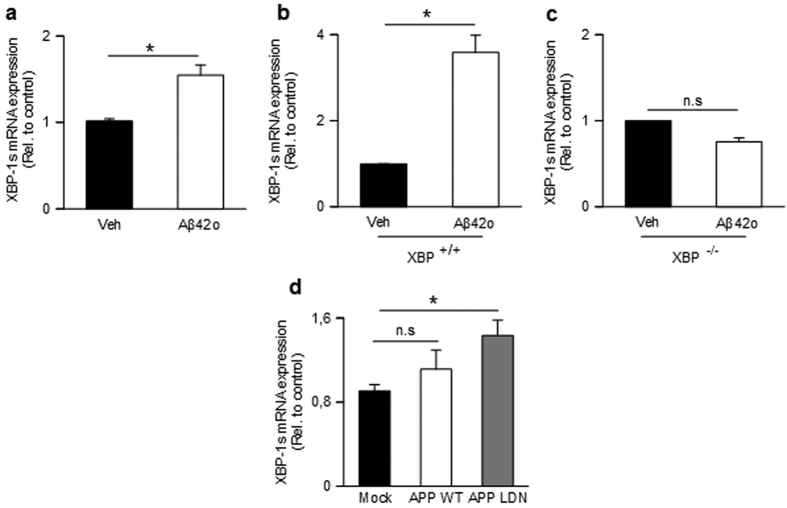
Influence of synthetic and natural Aβ42 oligomers on XBP-1s mRNA levels. (**a**) Quantitative PCR analysis of XBP-1s mRNA in SH-SY5Y (**a**), XBP^+/+^ (**b**) and XBP^−/−^ (**c**) cells treated overnight with synthetic Aβo. XBP-1s mRNAs are normalized using the expression of human Rpl69 housekeeping gene. The graphs show the mean of three independent experiments (6 and 3 biological replicates per group in (**a**,**b** and **c**), respectively). Student one-tailed T-test is applied for statistics (*p-value = 0,02 (**a**) and 0,03 (**b**,**c**); n.s non-significant). (**d**) Quantitative PCR analysis of XBP-1s mRNA in indicated CHO cell lines. XBP-1s mRNAs are normalized as above and graph shows the mean of 3 independent experiments (9 biological replicates per group). Statistical analysis is performed by a one-way ANOVA followed by a post hoc Bonferroni-Holm is applied (*p-value = 0,02 alpha 0,05; n.s, non-significant).

The intimate link between cellular stress, Aβ and BACE1^23^,^36^ led us to investigate whether Aβo effects on BACE1 expression could be governed by cellular sensors involved during the unfolded protein response in response to Aβ aggregation. Since Aβo affect BACE1 transcription, we envisioned XBP-1s, a transcription factor activated upon stress-associated Ire1α-mediated maturation, as a putative target. Interestingly, XBP-1s mRNA levels were increased upon synthetic Aβo treatment of SH-SY5Y cells (Fig. 2a). We ruled out the possibility of a cell-dependent artifact by monitoring Aβo-induced modulation of XBP-1s in murine fibroblasts. Thus synthetic Aβo also triggered enhanced XBP-1s mRNA levels in wild-type (Fig. 2b) but not in XBP-1s null fibroblasts (Fig. 2c). Of importance, we show that naturally occurring Aβo also increase XBP1s mRNA levels (Fig. 2d) even if we did not examine the precise nature of oligomers species involved in such effect. Overall, this data demonstrates the ability of both synthetic and naturally occurring monomers/Aβo mix to up-regulate XBP-1s mRNA levels.

### XBP-1s regulates BACE1 expression and activity at a post-transcriptional level

**Figure 3:**
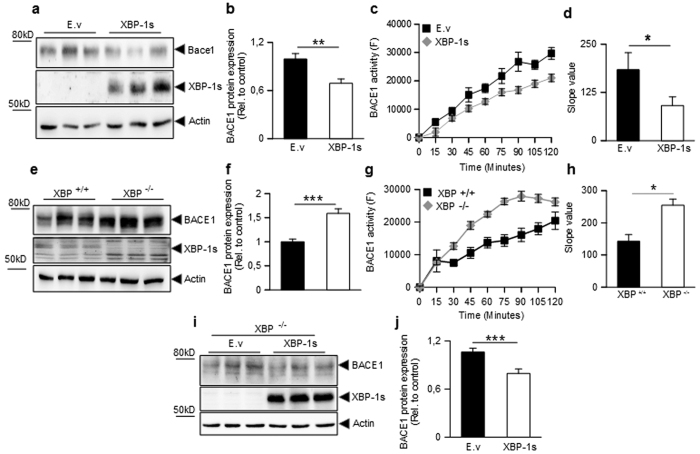
Modulation of BACE1 expression and catalytic activity by XBP-1s. (**a–h**) Western blot analysis (**a**,**e**), densitometric quantification (**b**,**f**) and specific activities (**c**,**g**) of BACE1 24 hours after transient transfection of HEK293 cells with XBP-1s encoding vector or empty vector (E.v) (**a–c**) or in XBP^+/+^ or XBP^−/−^ mouse fibroblasts (**e–g**). BACE1 activity is expressed relatively to control and normalized by protein quantification using Bradford method. Graphs represents the mean of 6 (**b**), 3 (**c**,**g**), and 5 (**f**) independent experiments and correspond to 24 (**b**), 9 (**c**) and 15 (**f**,**g**) biological replicates per group. In (**d** and **h**), bars represent the slope values of the curves presented in (**c** and **g**), respectively. All statistics are carried out with student one-tailed T-test: (**p-value = 0,0076 (B); *p-value = 0,04 (D); ***p-value = 0,0006 (**f**); *p-value = 0,04 (**h**). (**i,j**) Western blot analysis (**i**) and densitometric quantification (**j**) of BACE1 expression 24 hours after transient transfection of XBP^−/−^ cells with either control empty vector (E.v) or XBP-1s cDNA. Bars in (**j**) are the mean of 5 independent experiments (15 biological replicates per group). ***p-value = 0,0002.

We reasoned that since Aβo increase both BACE1 and XBP-1s, the latter could behave as an intermediate cellular effector mediating Aβo-associated increase in BACE1 transcription. Thus, we investigated the effect of a modulation of XBP-1s on BACE1 expression and protease activity. Surprisingly, we found that overexpression of XBP-1s reduces BACE1 expression (Fig. 3a,b) and enzymatic activity (Fig. 3c,d) in HEK293 cells. Conversely, XBP-1s depletion increases BACE1 expression (Fig. 3e,f) and activity (Fig. 3g,h), a phenotype that can be rescued by XBP-1s overexpression in XBP-1s knockout cells (Fig. 3i,j).

**Figure 4:**
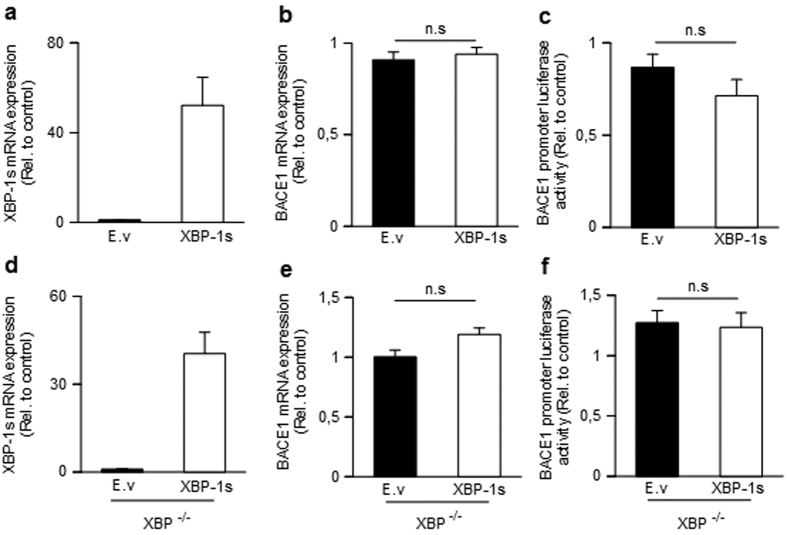
Influence of XBP-1s on BACE1 promoter transactivation and mRNA levels. (**a**,**b**) Quantitative PCR analysis of XBP-1s (**a**,**d**) and BACE1 (**b**,**e**) mRNA levels 24 hours after transient transfection of HEK293 (**a**,**b**) or XBP1^−/−^ (**d**,**e**) cells with either empty vector (E.v) or XBP-1s cDNA. BACE1 and XBP-1s mRNAs are normalized using the expression of Rpl69 housekeeping gene. (**c**,**f**) Luciferase activity measured in HEK293 (**c**) or XBP1^−/−^ (**f**) cells co-transfected with either empty vector (E.v) or XBP-1s cDNA, a BACE1 human promoter in frame with luciferase transcript (see methods) and a vector coding for beta-galactosidase. Luciferase activity was normalized with beta-galactosidase activity and protein concentration of samples and then expressed relatively to control. Graphs show the mean of four (**a**–**c**) or 3 (**d–f**) independent experiments corresponding to 24 (**a**,**b**), 12 (**c**) and 18 (**d–f**) biological replicates per group. Student one-tailed T-test is applied for statistics (n.s not significant).

We then examined whether XBP-1s-linked modulation of BACE1 expression and activity could be accounted for BACE1 promoter transactivation by XBP-1s. As expected, XBP-1s overexpression yields quantifiable amounts of XBP-1s mRNA (Fig. 4a) while it neither altered BACE1 mRNA levels (Fig. 4b) nor BACE1 promoter transactivation (Fig. 4c). To rule out any cell specific artifacts, these results were confirmed in fibroblasts genetically invalidated for XBP-1. As was observed in HEK293 cells, restoration of XBP-1s expression in null cells (Fig. 4d) did not modify BACE1 mRNA levels (Fig. 4e) or promoter transactivation (Fig. 4f). This post-transcriptional XBP-1s-mediated regulation of BACE1 was also observed in cells of neuronal origin (SH-SY5Y) (Supplementary Fig. 1a–c). Altogether, this set of data indicates that XBP-1s down-regulates BACE1 at a post-transcriptional level in both human neuroblastoma cells and mouse fibroblasts.

### XBP-1s regulates HRD1 expression at a transcriptional level

Our data indicate that Aβo increases BACE1 and XBP-1s but that the latter lowers BACE1 by post-transcriptional mechanisms. We thus reasoned that such cascade of events could only be explained by an intermediate effector between XBP-1s and BACE1 that should fulfill three criteria: (1) to be up-regulated by XBP-1s; (2) to act as a repressor of BACE1 and; (3) to trigger its effect at a post-transcriptional level.

**Figure 5:**
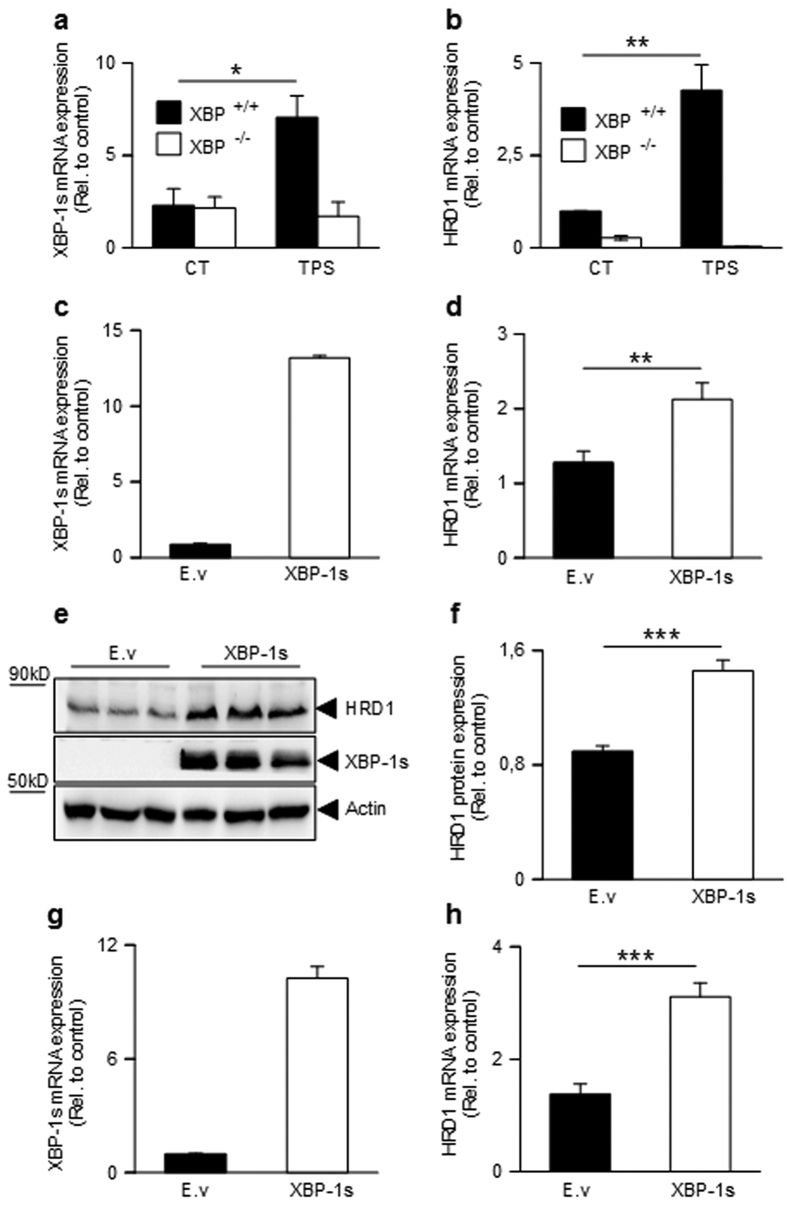
Modulation of HRD1 by thapsigargin and XBP-1s in human cells and XBP-depleted cells. (**a**,**b**) Quantitative PCR analysis of XBP-1s (**a**,**c**) and HRD1 (**b**,**d**) mRNA levels in XBP^+/+^ or XBP^−/−^ fibroblasts control (Ct) or treated 6 hours with thapsigargin (1 μM, TPS, (**a**,**b**) or 24 hours after transient transfection of HEK293 cells with empty vector (E.v.) or XBP-1s (**c**,**d**). (**e**,**f**) Western blot analysis (**e**) and densitometric quantification (**f**) of HRD1 protein expression in HEK293 cells transiently transfected with XBP-1s as above. (**g**,**h**) Quantitative PCR analysis of XBP-1s (**g**) and HRD1 (**h**) mRNA levels 24 hours after transient transfection of XBP^−/−^ cells with either empty vector (E.v.) or XBP-1s. All HRD1 and XBP-1s mRNAs are normalized using the expression of mouse Rpl69 housekeeping gene. Graphs shows the mean of 3 independent experiments, 6 (**a**,**b**), 18 (**c**,**d**,**g**,**h**) and 9 (**f**) biological replicates per condition. Student one-tailed T-test are applied for statistics (*p-value = 0,02 (**a**); **p-value = 0,01 (**b**); **p-value = 0,004 (**d**); ***p-value = 0,00001 (**f**); ***p-value 0,00007 (**h**)).

Yamamoto and Colleagues demonstrated that HRD1, an E3 ubiquitin ligase, was up-regulated under ER stress conditions through an XBP-1 dependent pathway^37^. This led us to envision HRD1 as the cellular effector linking XBP-1s to BACE1. First, we show that XBP-1s mRNA levels are enhanced in the presence of thapsigargin, a well known ER-stress inductor (Fig. 5a), and that this activation, as expected, is indeed fully abolished by XBP-1 genetic invalidation. HRD1 mRNA levels were also enhanced by thapsigargin and this increase was abolished by XBP-1s depletion (Fig. 5b). To strengthen this observation, we examined the influence of XBP-1s expression on HRD1 mRNA levels in human cells. As expected, XBP-1s overexpression enhances both HRD1 mRNA (Fig. 5d) and protein (Fig. 5e,f) expressions. These results were conforted by the ability of XBP-1s to increase HRD1 (Fig. 5h) in MEF cells devoid of XBP-1s (Fig. 5g). The effect of XBP-1s on HRD1 transcription was also confirmed in SH-SY5Y cells (Supplementary Fig. 1d). Overall, our results show that XBP-1s up-regulates HRD1 transcription in our cellular models.

### Aβ42 oligomers increase HRD1 expression through XBP1-dependent mechanisms

**Figure 6:**
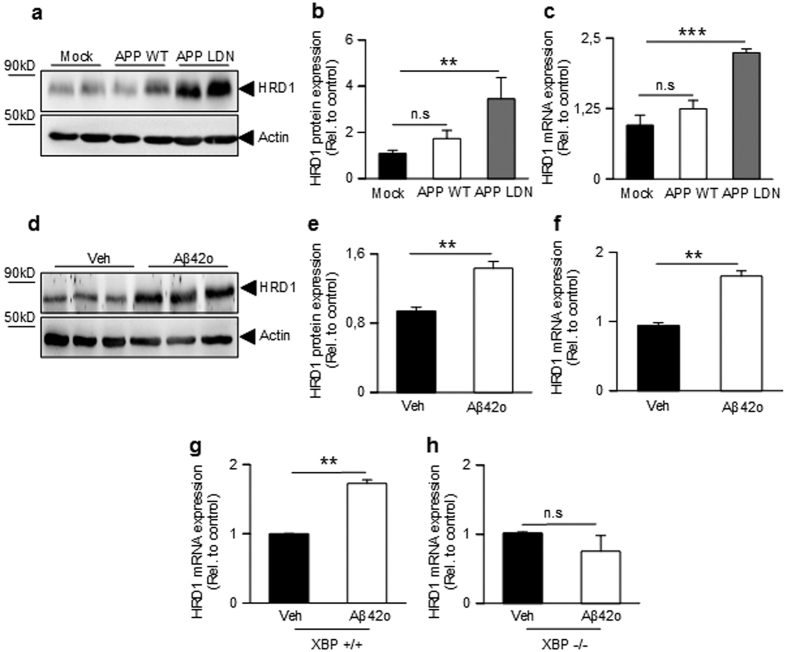
Effect of synthetic and natural Aβ42o on HRD1 protein and mRNA expressions and effect of Aβ42o on BACE1 in XBP^−/−^ fibroblasts. (**a–f**) Western blot (**a**,**d**), densitometric analysis (**b**,**e**) and mRNA levels (**c**,**f**) of HRD1 in indicated CHO cell lines (**a–c**) or in synthetic Aβo-treated SH-SY5Y (**d–f**). (**g**,**h)** Quantitative PCR analysis of HRD1 mRNA levels in Aβo-treated XBP^+/+^ (**g**) and XBP^−/−^ (**h**) cells. HRD1 mRNAs are normalized using the expression of Top1 housekeeping gene (CHO cell line) or Rpl69 (SH-SY5Y and fibroblasts). Graphs show the mean of 4 (**b**) or 3 (**c**,**e**–**h**) independent experiments corresponding to 10 (**b**), 6 (**c**,**f**), 8 (**e**) and 3 (**g**,**h**) biological replicates per group. In (**b** and **c**), a one-way ANOVA followed by a post hoc Bonferroni-Holm test is applied for statistics (**p-value = 0,01 alpha 0,05 (**b**) and ***p-value = 0,00000246 alpha 0,01 (**c**); n.s non significant). In (**e**–**h**), a student one-tailed T-test is applied for statistics (**p-value = 0,002 (**e**), **p-value = 0,01 (**f**); **p-value = 0,0047 (**g**); n.s: not significant).

Since Aβo increase XBP-1s that up-regulates HRD1, we assumed that a linear cascade should be reflected by an up-regulation of HRD1 by Aβo. We examined whether naturally secreted and synthetics Aβo indeed modulate HRD1 expression. Figure 6 shows that HRD1 protein (Fig. 6a,b) and mRNA (Fig. 6c) levels were enhanced in CHO APP_LDN_ cells. This data was corroborated by the fact that synthetic Aβo also enhanced HRD1 protein (Fig. 6d,e) and mRNA levels (Fig. 6f). Of most interest, Aβo-associated increased amount of HRD1 mRNA expression in wild-type fibroblasts (Fig. 6g), was fully abolished by XBP1 gene invalidation (Fig. 6h). Thus, this data demonstrates a direct link between Aβo-mediated increases in XBP-1s and HRD1.

### HRD1 reduces BACE1 expression

**Figure 7:**
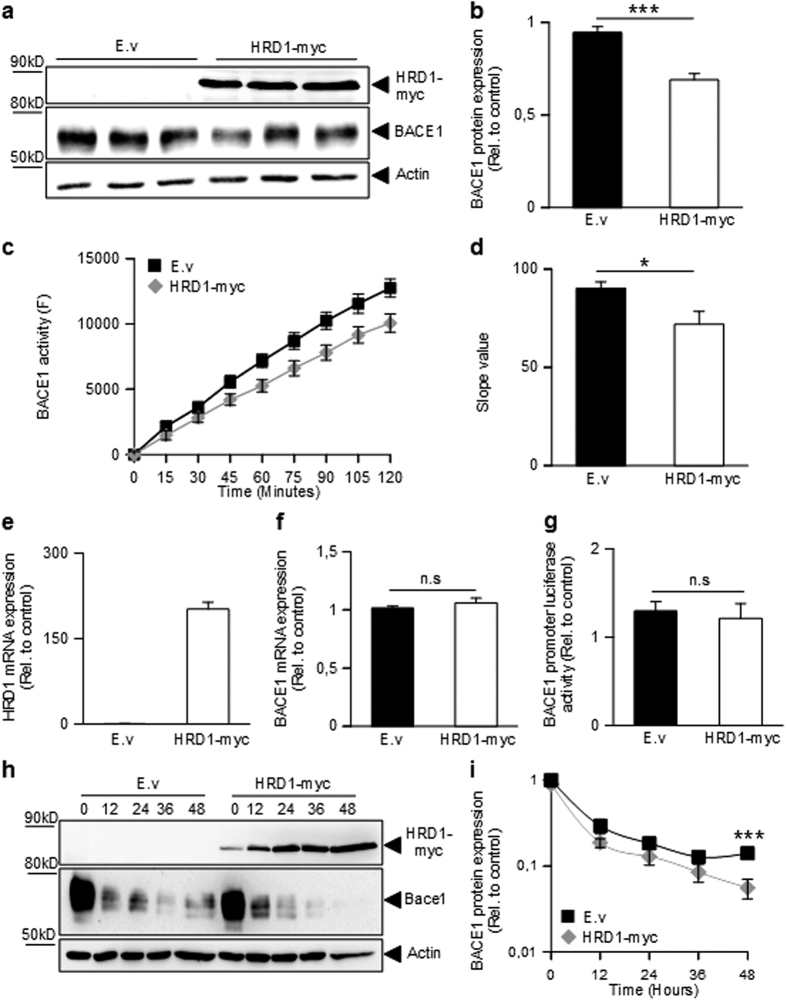
HRD1 controls BACE1 at a post-transcriptional level. Western blot analysis (**a**), densitometric quantification (**b**) and specific activities (**c**) of BACE1 24 hours after transient transfection of HEK293 cells with control (E.v) or HRD1-myc cDNA. BACE1 activity is expressed relatively to control and normalized by protein quantification using Bradford method. Graphs represents the mean of 6 (**b**) and 3 (**c**) and correspond to 18 (**b**) and 9 (**c**) biological replicates per group. (**d)** Represents the slope value of the curve presented in (**c**). Student one-tailed T-test is applied for statistics (***p-value = 7 × 10^−8^ (**b**) and *p-value = 0,03 (**c**). (**e**,**f**) Quantitative PCR analysis of HRD1 and BACE1 mRNA 24 hours after transient transfection of HEK293 cells with control (E.v) or HRD1-myc cDNA. BACE1 and HRD1 mRNAs are normalized using the expression of human Rpl69 housekeeping gene. The graph shows the mean of 3 independent experiments (9 biological replicates per group). Student one-tailed T-test is applied for statistics (n.s non significant). (**g**) Luciferase activity measured in HEK293 cells co-transfected with control or HRD1 coding vector, a BACE1 human promoter in frame with luciferase and a vector coding for beta-galactosidase. Luciferase activity was normalized with beta-galactosidase activity and protein concentration of samples and then expressed relatively to control. The graph shows the mean of 3 independent experiments (18 replicates per group). Student one-tailed T-test is applied for statistics (n.s non significant). (**h**) Western blot analysis of HEK293 cells transiently transfected with control (E.v.) or HRD1-myc cDNA for 12 h before treatment with cycloheximide (1 μM) up to 48 h. (**i**) Densitometric analysis of BACE1 expression normalized with actin and represented relatively to control (cells transfected with control vector at T0) through time. Note that ordinate axis follows a logarithmic scale. The graph shows the quantification of four independent experiments (4 replicates per condition). Student one-tailed T-test is applied for statistics (***p-value = 0,001).

To validate the molecular cascade linking Aβo-mediated increase of XBP-1s and HRD1 to XBP-1s-associated decrease of BACE1, one should envision HRD1 as an intermediate down-regulator of BACE1. Figure 7 shows that it was indeed the case. Thus, HRD1 overexpression decreased BACE1 protein expression (Fig. 7a,b) and activity (Fig. 7c,d) by about 30%. However, HRD1 expression (see increased HRD1 mRNA expression in Fig. 7e) was unable to modulate BACE1 promoter transactivation (Fig. 7f) and mRNA levels (Fig. 7g). Thus, HDR1-linked down-regulation of BACE1 likely occurs at a post-transcriptional level. This was supported by the reduced lifetime of BACE1 upon HRD1 expression (Fig. 7h,i) in experimental conditions where neosynthesis of endogenous BACE1 was blocked by cycloheximide (see Methods), in agreement with the HRD1 function as an ubiquitin ligase^38^. Overall, the above-described data demonstrate a linear molecular cascade by which Aβo decrease BACE1 via an XBP-1s-mediated and HRD1-dependent mechanism.

### Additional XBP-1s-independent control of BACE1 by Aβo oligomers

**Figure 8:**
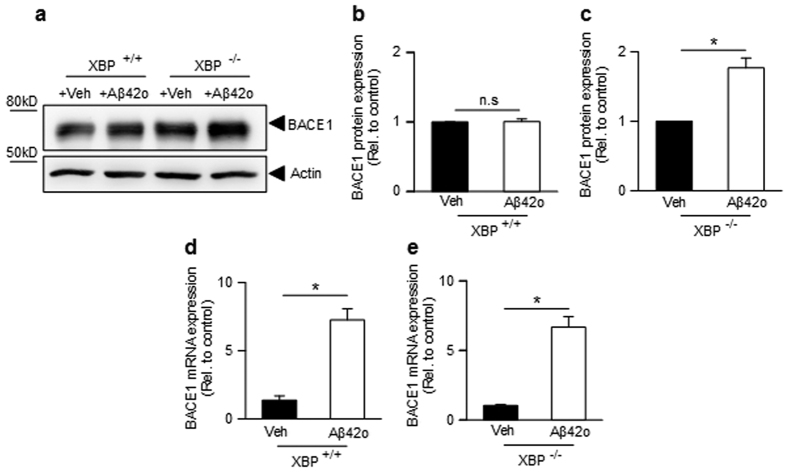
Influence of XBP1 depletion on Aβ42o-induced effect on BACE1 protein and mRNA expression. (**a**–**c**) Western blot analysis (**a**), densitometric quantification (**b**,**c**) and quantitative PCR mRNA analysis (**d,e**) of BACE1 after an overnight treatment of XBP^+/+^ (**a**,**b**,**d**) or XBP^−/−^ (**a**,**c**,**e**) fibroblasts. BACE1 mRNAs are normalized using the expression of human Rpl69 housekeeping gene. Bars show the mean of 3 independent experiments (3 biological replicates per group). Student one-tailed T-test is applied for statistics (*p-value = 0,03 (**c**); *p-value = 0,03 (**d**); *p-value = 0,02) (**e**).

At first sight, the above data linking Aβo to a decrease of BACE1 appear contradictory to our initial observation that both synthetic and naturally occurring Aβo increased BACE expression and activity (see Fig. 1). This led us to question whether Aβo could modulate BACE1 exclusively via XBP-1s or if there exist alternative pathway by which Aβo could control BACE1. Thus, we examined the influence of synthetic Aβo on BACE1 in absence of XBP-1s. Figure 8a–e clearly shows that Aβo could still increase BACE1 expression in XBP-1s depleted cells (Fig. 8a–c). Interestingly, this appeared to occur at a transcriptional level since Aβo also increased BACE1 mRNA levels in XBP^−/−^ cells (Fig. 8e). This indicates that besides, XBP-1s-mediated HRD1-linked post-translational events aimed at reducing BACE1, Aβo could also increase BACE1 by alternative XBP-1s-independent transcriptional pathway. Thus, both Aβo-linked XBP-1s- dependent and -independent pathways could occur and one can envision that the former likely aims at preventing acute Aβo-induced toxicity and increased BACE-1-associated self-production, at least at early stages of the pathology.

## Discussion

Numerous biochemical, anatomical and genetic data led to the claim that the so-called amyloid cascade hypothesis fulfils the major gap in the understanding of Alzheimer’s disease (AD) etiology. For years, it has been proposed that senile plaques that accumulate in AD-affected brains correspond to the pathological lesions and thus, their main components, amyloid β-peptides (Aβ) thought to be at the center of gravity of the neurodegenerative process, drove much attention. This simplistic view of a complex disease has recently drastically evolved. Besides altered ratios in the canonical forms of Aβ40 and Aβ42 that are indeed affected in AD brain^39^, recent advances on the structural nature (N-terminally truncated and/or C-terminally trimmed^40^,^41^, biophysical state (monomeric, multimeric, aggregated, fibrillar^34^,^42^, subcellular localization (intracellular, secreted^43^,^44^) of Aβ species as well as other βAPP catabolites (C99, AICD^5^,^11^,^12^,^13^,^45^, have significantly modified our view of the genuine trigger of the pathology. It arose from these studies that soluble Aβ oligomers (Aβo), appear prior to senile plaques and are now considered to be more toxic than monomeric or fibrillar Aβ. Supporting this view, amongst a series of cellular perturbations, Aβo contribute to neuronal cell death, LTP inhibition^46^ calcium homeostasis perturbation^47^, oxidative or ER stresses^48^.

Whatever the toxic Aβ-related trigger, it remains that understanding the upstream enzymatic steps ultimately yielding Aβ was of major importance. Although the nature of the secretases is consensual, the mechanisms by which their activity is finely tuned or regulated remained a matter of investigation.

The choice of the secretase as a therapeutic target is a hard one. Most attention was originally centered on γ-secretase because this enzyme triggers the ultimate cleavage yielding Aβ. However, γ-secretase-mediated breakdowns of numerous additional substrates involved in vital cellular functions^49^ have been documented and it has been hard to design specific inhibitory compounds usable in clinic. This likely explains reiterated failures of γ-secretase-centric clinical trials and has severely tempered the optimism for considering γ-secretase blockade as a mean to interfere with the course of the disease. Although BACE1 was recently shown to contribute to several physiological functions^50^,^51^,^52^,^53^,^54^, it appears that unlike is the case for γ-secretase^55^,^56^. BACE1 gene ablation is rather well supported in animals, indicating a more narrow substrates specificity than that of γ-secretase or, alternatively, that functions of its additional substrates appear less vital for cells. These considerations suggest that the targeting of BACE1 appears apparently less challenging. Furthermore, this strategy presents several advantages. First BACE1 is the rate-limiting enzyme of Aβ biosynthetic pathway. Second, the blockade of BACE1 not only impairs Aβ production but also theoretically prevents all βAPP-related catabolites, some of which appear very toxic, even in absence of Aβ^11^,^12^,^13^.

Relatively few data concern BACE1 regulation and most of them linked Aβ load or βAPP mutations to altered BACE1 gene transcription^16^,^17^,^32^. More often, BACE1 expression or activity have been linked to cellular conditions mostly related to hypoxia^36^,^57^, oxidative^58^ and ER stress^23^,^59^.

XBP-1 is a transcription factor known to regulate genes involved in ER homeostasis that has also been involved in multiple signaling pathways and diseases (For review see ref. 28). Here we show that XBP-1s lowers BACE1 expression and activity in various cells of human and murine origins. Interestingly, XBP-1s-mediated repression of BACE1 occurs at a post-transcriptional level. This agrees well with a recent study showing that XBP1s did not modify BACE1 promoter transactivation in human cells^60^. In search for a cellular intermediate, we reasoned that it should be either a BACE1 activator that would be transcriptionally repressed by XBP-1s or, alternatively, a BACE1 repressor, the transcription/activity of which would be enhanced by XBP-1s. The latter case stood. Thus we demonstrated that HRD1 mRNA levels were up-regulated by XBP-1s and that HRD1 down-regulated BACE1 protein but not mRNA expressions. This agreed with the well-documented function of HRD1 that acts as an ubiquitin ligase involved in protein ubiquitination and degradation during Endoplasmic Reticulum associated degradation (ERAD), a process known to be under control of XBP-1^61^. The above-described set of data was comforted by the fact that Aβo increased both XBP1s and HRD1 expressions. This agreed with several independent studies showing that XBP-1 mRNA splicing, and therefore activation, was potentiated by Aβo in transgenic flies^30^ and primary cultured neurons^62^ see Suppl. Fig. 12) and demonstrating an Aβo-mediated post-transcriptional regulation of neuronal BACE1-like immunoreactivity^63^. In addition, previous works indicated a negative correlation between HRD1 expression and Aβ generation^64^ and demonstrated that HRD1 suppression leads to enhanced Aβ production^65^,^66^.

We propose that Aβo-mediated increase in XBP-1s could be seen as a compensatory mechanism aimed at down-regulating BACE1 activity and thus, interfering with a potential vicious cycle by which Aβo feed their own production. This could occur to counteract Aβ-mediated increase in BACE1 activity by transcription factors such as c-Jun^16^, nuclear factor-kappa B (NF-κB)^17^, nuclear factor of activated T-cells 1 (Nfat-1)^18^, specificity protein 1 (Sp1)^19^, Yin Yang 1 (YY1)^20^, and signal transducer activator of transcription (STAT3)^21^ as has been previously documented. Indeed, our study also clearly shows that Aβo could enhance BACE1 protein and mRNA expressions in XBP1 knockout cells (see Fig. 8).

The XBP-1S-mediated compensatory mechanism would agree with the protective phenotype generally ascribed to XBP-1s. Thus, previous studies showed that XBP-1s protected against Aβ-associated toxicity^30^ and could interfere with autophagy process^67^. More importantly and related to Alzheimer’s disease, XBP1 depletion revealed memory defects in wild-type mice^29^ and XBP-1s expression restored synaptic plasticity and memory control in several AD mice models^62^. Finally, XBP-1S transcriptionally up-regulates ADAM10, the α-secretase constitutive activity involved in the protective non amyloidogenic βAPP processing pathway^60^.

According to the time frame of AD development, we anticipate that compensatory mechanisms should occur early and transiently. This notion of temporal window is supported by studies on the expression of XBP1 in various AD mice models and AD-affected human brains. Thus, proteomic analysis revealed enhanced XBP1 protein expression at an early stage of disease progression in 5xFAD mice^68^. Further, Reinhard and colleagues showed that XBP-1s mRNA levels peaked at 8 months of age in APP/PS1^60^. Finally, we observed a transient increase of XBP-1s mRNA in the hippocampus of 3 month-old CRND8 and 3xTg-AD mice^62^. Interestingly, XBP-1s expression was lowered in the frontal and temporal cortices in autopsied AD affected brains, i.e at late stage of the disease, when BACE1 expression has been clearly shown to be enhanced^14^. This is consistent with the claim of a protective increase in XBP1 aimed at reducing BACE1 expression at early stage of the pathology, a transient compensatory process ineffective at later stage of the pathology.

Gene therapy has been proposed as a track to target brain pathologies linked to ER stress^69^. In this context, it is noteworthy that modulating XBP1 has proved efficient to protect against Huntington disease in mice model of this pathology. Whether XBP-1s targeting would be efficient in the case of Alzheimer’s disease remains a matter of speculation and still awaits faithful biomarkers that could tag the very early stages of the disease. However, it remains that our study shows that compensatory mechanisms should not be underscored and underlines the need for further understanding of secretases regulation.

## Methods

### Plasmids

The spliced form of mouse XBP-1 cDNA (XBP-1s) was cloned in pcDNA3 plasmid. The human HRD1-myc cDNA (transcript variant 2) was cloned in pCMV6 and obtained from OriGene Technologies. Luciferase construct of human BACE1 promoter cloned in pGL3-basic vector was kindly provided by Dr. Lahiri D.K.

### Cell culture and transfections

Human embryonic kidney cells (HEK293), human neuroblastoma cells (SH-SY5Y) and mouse embryonic fibroblasts (MEF) were cultured at 37 °C humidified air with 5% CO2 in Dulbeccos’s modified Eagle’s medium (4,5 g/L glucose) supplemented with fetal calf serum (10%). No antibiotics were added. Chinese ovary hamster cells (CHO) stably expressing wild type APP (APP_WT_) and APPV717I mutation (APP_LDN_), were obtained as described^35^ and cultured in Dulbeccos’s modified Eagle’s medium (4,5 g/l glucose) supplemented with fetal calf serum (10%), HT supplement (Gibco) and D-Proline. Transfections were performed with lipofectamine 2000 (Invitrogen) for mouse embryonic fibroblast (MEF) XBP^+/+^ and XBP^−/−^. Cells (about 300.000) were plated 24 h before transfection on 6 well plates, then DMEM medium was replaced by Dulbecco’s Optimem medium with a transfection mix (5 μl lipofectamine, 2,5 μg cDNA/well) for 6 hours. Other cell types were plated at about 500.000 cells per well and transfected with Jetprime (Polyplus-transfection) following manufacturer’s instruction. Harvest and analysis of cells were carried out 24 h after transfection.

### Protein extraction and western blotting

Cultured cells were collected in PBS-EDTA (5 mM) before centrifugation at 3,000 rpm for 5 min. Pellets were lysed in RIPA buffer with a complete proteases inhibitor, then centrifuged at 13,000 rpm for 10 min. Total protein concentration was assessed in the supernatant by Bradford method. Each sample (50 μg) was resuspended in Laemli buffer (1x final) and boiled at 96 °C for 5 min before loading and resolving on 10% SDS-PAGE gel. Wet transfer was done using Hybond-C membrane (Amersham Bioscience) at 100 V for 1 h30, then membrane were blocked with 5% PBS-milk solution for 1 h15 and blotted overnight with following antibodies: Mouse monoclonal 3D5 anti-Bace1 (kindly given by Dr. Vassar R.), rabbit polyclonal anti-XBP-1 (M-186 Santa-Cruz Biotechnology), mouse-monoclonal anti-actin (Sigma Aldrich), mouse-monoclonal anti-Myc (9E10; Santa-Cruz biotechnology). Protein immunoreactivity was assayed using peroxidase-coupled antibodies (Jackson immunoresearch), resulting electrochimio-luminescence was detected with luminescence analyzer LAS-3000 (Raytech). Multi-Gauge software (FUJI film) was used for image protein quantification. All densitometric quantifications were normalized using actin as loading control.

### RNA extraction and Quantitative Real-time PCR

Total RNA extractions were performed with RNA easy extraction kit (Qiagen) on Qiacube device following manufacturer’s material and methods. RNA quantities were normalized at 2000 ng and reverse-transcripted with GoScript reverse-transcriptase (Promega) on a Biometra thermocycler. Real time quantitative PCR were performed on Rotor-gene6000 (Qiagen) using SYBR Green protocol according to manufacturer’s recommendation. Gene expression of BACE1, XBP-1s and HRD1 were normalized using human or mouse ribosomal protein 69 (Rpl 69) gene expressions depending on RNA species. RNA relative expression was calculated following Livak K.J. delta-delta CT method^70^. Selected primers are listed below: cTOP Forward (F) 5′-AGG-ATC-ACA-GTG-GCT-TGG-TG-3′/Reverse (R) 5′-TCG-TAG-TCC-CGT-CGG-TCA-T-3′; cHRD1: F5′-AGG-TGT-CTT-ACC-CCC-GAA-GT-3′/R′-GGT-AGT-AGG-CAT-GAG-CCA-CC-5′; cXBP-1s: F5′-GAG-CTG-GAA-CAG-CAA-GTG-GT-3′/R 5′-GCCTGCACCTGCTGCG-3′; hTOP: F 5′-CCC-TGT-ACT-TCA-TCG-ACA-AGC-3′/R 5′-CCA-CAG-TGT-CCG-CTG-TTT-C-3′; hRPL69: F 5′-GGG-CAT-AGG-TAA-GCG-GAA-GG-3′/R 5′-TCA-GGT-ACA-GGC-GTG-GAT-ACA-3′; hXBP-1s: F 5′-AGC-TTT-TAC-GGG-AGA-AAA-CTC-A-3′/R 5′-ACA-GTC-GTC-TTG-GGACGT-G-3′; hHRD1: F 5′-GGC-AAC-AGG-AGA-CTC-CAG-CTT-3′/R 5′-CTG-CTT-CTG-CCA-CAG-CAT-C-3′; hBACE1: F 5′-ACA-CCA-GCT-GCT-CTC-CTA-GC-3′/F 5′-TGC-AGT-CAA-ATC-CAT-CAA-GG-3′; mRPL69: F 5′-CTG-ATC-AGG-GAT-GGG-CTG-AT-3′/F 5′-GCC-GCT-ATG-TAC-AGA-CAC-GA-3′; mXBP-1s: F 5′-AGC-TTT-TAC-GGG-AGA-AAA-CTC-A-3′/R 5′-GCC-TGC-ACC-TGC-TGC-G-3′; mHRD1: F 5′-TCT-GTG-CAG-CTG-GTA-TTT-GG-3′/R 5′-GGC-AAA-GAG-TGG-GAA-TGT-GT-5′; mBACE1: F 5′-TCC-TTC-CGC-ATC-ACC-ATC-3′/R 5′-ACA-GTC-GTC-TTG-GGA-CGT-G-3′.

### β-secretase enzymatic activity assay

Cells were harvested in PBS-EDTA 5 mM and centrifuged at 3,000 rpm for 5 min. After centrifugation, pellets were lysed on ice with an homogenization buffer (5 mM EDTA, 1 mM Hepes, 0,25 M sucrose) using a 26G syringe. The samples were then centrifuged at 850 g for 10 minutes and the supernatants were centrifuged once more at 20,000 g for 1 h. All preparations were normalized to a protein concentration of 3 μg/μl after Bradford quantification using Tris buffer (Tris 10 mM pH 7.5). 10 μl of each sample were incubated in a 96 wells plate with 90 μl acetate buffer (25 mM, pH 4.5) in presence or absence of BACE1 specific inhibitor (PromoKin). After 5 minutes of incubation, BACE1 fluorimetric substrate [(7-methoxycoumarin-4-yl)-acetyl-SEVNLDAEFRK(2,4-dinitrophenyl)-RRNH_2_; 10 μM R&D Systems] was added and the BACE1-like activity was monitored as described^71^ every 15 minutes for 2 h30 (320 nm excitation wavelength and 420 nm emission wavelength). Specific BACE1 activity was considered as the inhibitor-sensitive fluorimetric activity.

### Luciferase and β-galactosidase activities

Luciferase activities (reporting BACE1 promoter activities) were measured after cells co-transfection with both a luciferase reporter construct and a β-galactosidase encoding construct. Cells were lysed, on ice, using a reporter lysis buffer (Promega) before centrifugation at 2,500 rpm for 10 minutes. Supernatant fraction (20 μl) was mixed with luciferase reagent (50 μl) and luminescence was then measured on Varioskan flash reader (Thermo Scientific). Luciferase activity was normalized with either beta-galactosidase activity and protein concentration determined by Bradford method.

### Cycloheximide pulse-chase experiment

Cells were transfected with construct encoding HRD1-myc or a control empty vector for 12 hours. Cycloheximide (Sigma-Aldrich) was added to the cell media at a concentration of 1 μM up to 48 hours of time. Cells were harvested every 12 hours in PBS-EDTA buffer and centrifuged at 3,000 rpm for 5 minutes. Protein extraction and western blot were then carried out as indicated in the corresponding section.

### Synthetics Aβ42 oligomers preparation

Synthetic Aβ42 (Bachem Distribution Services GmbH, Weil am Rhein, Germany) was solubilized in HFIP (hexafluoro-2-propanol) according to previously described (Clarke *et al.*, 2015). Briefly, after solubilization and solvent evaporation, dried films were dissolved and stired in 10 μl dimethylsulfoxide. After dilution into 500 μl of cold PBS during 24 hours, insoluble aggregates were removed by centrifugation at 14,000 g for 10 min at 4 °C as described (De Felice *et al.*^48^). Supernatant was then stored at 4 °C. For each cell treatement, 300 μl of the preparation were used.

### Statistical analysis

Statistical analyses were performed with the Excel software. For statistical comparison between two groups, the parametric Student T-test (two tailed) was used. For statistical comparison between three groups, a one-way ANOVA test was performed, followed by the Bonferroni-Holme post hoc correction for multiple comparisons. Graphs represents the mean of the values obtained for each group of samples (biological replicates consisting of independent cells cultures, technical replicates were used in quantitative PCR as internal control for accuracy but are excluded from statistical analysis), error bars represent the standard error of the calculated mean (s.e.m). Criteria for data exclusion were: high background noise and low or heterogeneous specific signal, important loading control variation (Western blot), important modulation of housekeeping gene expression or low accuracy between technical replicates (quantitative PCR), unequal β-galactosidase activity between samples (Luciferase dosage), flat specific enzymatic curve with no exponential phase (β-secretase enzymatic activity assay).

## Additional Information

**How to cite this article**: Gerakis, Y. *et al.* Aβ42 oligomers modulate β-secretase through an XBP-1s-dependent pathway involving HRD1. *Sci. Rep.* **6**, 37436; doi: 10.1038/srep37436 (2016).

**Publisher’s note:** Springer Nature remains neutral with regard to jurisdictional claims in published maps and institutional affiliations.

## Supplementary Material

Supplementary Information
