# Supplementary material for: Aβ42 oligomers modulate β-secretase through an XBP-1s-dependent pathway involving HRD1
Source: Sci Rep. 2016 Nov 17;6:37436. doi: 10.1038/srep37436 (PMC5112606; doi:10.1038/srep37436)
Supplement: Supplementary Information [file srep37436-s1.pdf]

Supplementary figure

Aβ42 oligomers modulate β-secretase through an XBP-1s-dependent pathway involving HRD1

Yannis Gerakis[1], Julie Dunys[1], Charlotte Bauer[1] and Fréderic Checler[1*]

[1] Université Côte d'Azur, INSERM, CNRS, IPMC, France, Laboratory of excellence DistALZ, 660 route des Lucioles, 06560, Sophia-Antipolis, Valbonne, France

* Corresponding author.
Correspondence and material requests should be addressed to: checler@ipmc.cnrs.fr

Legends

Supplementary figure 1 : Modulation of BACE1 and HRD1 expression by XBP-1s in SH-SY5Y cells.
a, b: Western blot analysis (**a**), densitometric quantification (**b**) of BACE1 24h after transfection of SH-SY5Y cells with either empty vector (E.v) or XBP-1s encoding vector. **c, d**: Quantitative PCR mRNA analysis of BACE1 mRNA (**c**) or HRD1 mRNA (**d**) 24h after transfection of SH-SY5Y cells with either empty vector (E.v) or XBP-1s encoding vector. mRNAs expression is normalized using the expression of Rpl69 housekeeping gene. Bars show the mean of 4 independent experiments (3 biological replicates per group). Student one-tailed T-test is applied for statistics (***p-value = 0,001 (**a**); n.s, non-significant (**c**) ***p-value = 0,0012 (**d**))

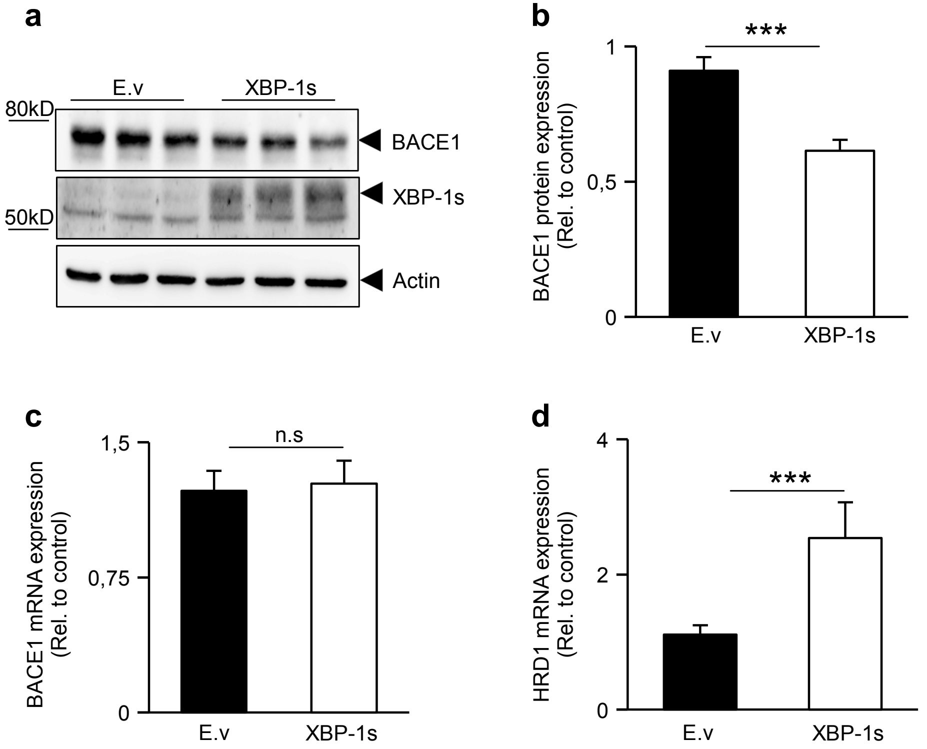

Supplementary figure 1. Gerakis et al.
